# Targeting SPARC by lentivirus-mediated RNA interference inhibits cervical cancer cell growth and metastasis

**DOI:** 10.1186/1471-2407-12-464

**Published:** 2012-10-10

**Authors:** Jie Chen, Dehuan Shi, Xiaoyan Liu, Shuang Fang, Jie Zhang, Yueran Zhao

**Affiliations:** 1 Department of Maternal and Child Health Care, School of Public Health, Shandong University, Jinan, 250012, China; 2 Department of Obstetrics and Gynecology, Qilu Hospital, Shandong University, Jinan, 250012, China; 3 Major of Clinical Medicine, Preclinical Medicine College, Taishan Medical University, Taian, 271000, China; 4 Central Laboratory, Shandong Provincial Hospital affiliated to Shandong University, Jinan, 250021, China

**Keywords:** SPARC, Cervical cancer, Proliferation, Apoptosis, Metastasis

## Abstract

**Background:**

Secreted protein acidic and rich in cysteine (SPARC), a calcium-binding matricellular glycoprotein, is implicated in the progressions of some cancers. However, no information has been available to date regarding the function of SPARC in cervical cancer cell growth and metastasis.

**Methods:**

In this study, we isolated and established high invasive subclones and low invasive subclones from human cervical cancer cell lines HeLa and SiHa by the limited dilution method. Real-time q-RT-PCR, Western Blot and ICC were performed to investigate SPARC mRNA and protein expressions in high invasive subclones and low invasive subclones. Then lentivirus vector with SPARC shRNA was constructed and infected the highly invasive subclones. Real-time q-RT-PCR, Western Blot and ICC were also performed to investigate the changes of SPARC expression after viral infection. In functional assays, effects of SPARC knockdown on the biological behaviors of cervical cancer cells were investigated. The mechanisms of SPARC in cervical cancer proliferation, apoptosis and invasion were also researched.

**Results:**

SPARC was over-expressed in the highly invasive subclones compared with the low invasive subclones. Knockdown of SPARC significantly suppressed cervical cancer cell proliferation, and induced cell cycle arrest at the G1/G0 phase through the p53/p21 pathway, also caused cell apoptosis accompanied by the decreased ratio of Bcl-2/Bax, and inhibited cell invasion and metastasis accompanied by down-regulated MMP2 and MMP9 expressions and up-regulated E-cadherin expression.

**Conclusion:**

SPARC is related to the invasive phenotype of cervical cancer cells. Knockdown of SPARC significantly suppresses cervical cancer cell proliferation, induces cell apoptosis and inhibits cell invasion and metastasis. SPARC as a promoter improves cervical cancer cell growth and metastasis.

## Background

Cervical cancer is the second most common malignancy in women worldwide, and it remains a leading cause of cancer-related death for women in developing countries [1]. About 30% of patients with International Federation of Gynecology and Obstetrics (FIGO) stage IB2 to stage IV will ultimately recur and metastasis [2]. Therefore, studying the mechanisms of tumor invasion and metastasis will provide further insights into the occurrence and development of cervical cancer. In recent years, many genes, such as the high mobility group box l [3], metastasis-associated 1 [4], Twist [5], and so on, have been found correlated with the progression of cervical cancer. However, few studies have been done to assess the functions of SPARC in cervical cancer cell growth and metastasis.

SPARC, also termed osteonectin, BM-40, and 43K protein, was initially identified by Termine et al. [6] as a bone-specific phosphoprotein that binds to collagen fibrils and hydroxyapatite at distinct sites. SPARC has generated considerable interests as a multi-faceted protein that belongs to a family of matricellular proteins, whose function is to modulate cell matrix interactions and cell function [7]. Recently, SPARC has been found over-expressed in a variety of cancers and considered a potential target for cancer therapy [8,9], but the function of SPARC in cervical cancer cell growth and metastasis is not fully understood.

In the present study, we isolated and established high invasive subclones and low invasive subclones from human cervical cancer cell lines HeLa and SiHa by the limited dilution method. We found that the expressions of SPARC in high invasive subclones were much higher than that in low invasive subclones. In the function assays, we decreased the expressions of SPARC in high invasive subclones by lentivirus-mediated RNA interference to determine the effects of SPARC on cervical cancer cell proliferation, apoptosis, invasion and metastasis. To our knowledge, it is the first time to clarify the function and mechanism of SPARC in cervical cancer cell growth and metastasis.

## Methods

### Cell lines

HeLa and SiHa cervical cancer cell lines were obtained from Shanghai Institute for Biological Sciences, Chinese Academy of Sciences. The cervical adenocarcinoma cell line HeLa containing HPV18 DNA, has a small telocentric chromosome in 98% of the cells and 100% aneuploidy. The cervical squamous carcinoma cell line SiHa containing HPV16 DNA, is a hypertriploid human cell line with the modal chromosome number of 71, occurring in 24% of cells. Cells were cultured in DMEM supplemented with 10% fetal bovine serum and antibiotics (Gibco BRL, Rockville, MD).

### Isolation of HeLa and SiHa cell subclones

Cells at log phase were collected and adjusted to a density of 10 cells/ml with RPMI-1640, then seeded into a 96-well plate (0.1 ml/well). The 96-well plate was observed through a microscope, and then a marker was made on the well containing a single cell. After being cultured for 2–3 weeks at 37°C and 5% CO_2_ condition, a good clone was selected and amplified. A total of 25 cell subclones were obtained from HeLa cells and named from HeLa-1 to HeLa-25, meanwhile a total of 23 subclones were obtained from SiHa cells and named from SiHa-1 to SiHa-23. The electrophoretic migration rates of these subclones were measured by the cell electrophoretic instrument (DY-100, from College of Life Science, Shandong University, China). A total of 15 cells /subclone were measured and the averages were figured out. All data were expressed as mean ± SE.

### RNA interference

Self-inactivating lentivirus vector (GeneChem, Shanghai, China) containing a CMV-driven GFP reporter and a U6 promoter upstream of the cloning sites (Age I and EcoR I) was used for cloning small hairpin RNAs (shRNAs). The target sequence for SPARC was 5^′^- AACAAGACCTTCGACTCTTCC-3^′^; the negative control sequence was 5^′^-TTCTCCGAACGTGTCACGT-3^′^. HeLa-1 and SiHa-1 cells were cultured in six-well tissue culture plates and infected with lentivirus at a multiplicity of infection (MOI) of 60 for 24 h. Then the medium was replaced with fresh complete medium. After 4 days, cells were observed under fluorescence microscopy to confirm that more than 80% of cells were GFP-positive.

### Real-time quantitative RT-PCR (q-RT-PCR)

Total RNA was extracted using Trizol reagent (Invitrogen) and reversed transcribed. Quantitative real-time PCR analysis was performed using ABI PRISM 7500 Real-Time PCR System (Applied Biosystems). Each well (20 μl reaction volume) contained 10 μl Power SYBR Green PCR master mix (Applied Biosystems), 1 μl of each primer (5 μmol/l) and 1μl template. The primers (Table 1) were designed by primer 5 software and synthesized by TaKaRa Biotechnology (Dalian) Co., Ltd.

**Table 1:** Real time RT-PCR primers

**Genes**	**Forward and reverse primer**	**Product length (bp)**
SPARC	F: 5^′^-ACATAAGCCCAGTTCATCACCA-3^′^	278
	R: 5^′^-ACAACCGATTCACCAACTCCA-3^′^	
E-cadherin	F: 5^′^-GGATTGCAAATTCCTGCCATTC-3^′^	147
	R: 5^′^-AACGTTGTCCCGGGTGTCA-3^′^	
β-catenin	F: 5^′^-GCTGATCTTGGACTTGATATTGGTG -3^′^	117
	R: 5^′^- GTCCATACCCAAGGCATCCTG -3^′^	
α-catenin	F: 5^′^- CTCTACTGCCACCAGCTGAACATC -3^′^	154
	R: 5^′^- ATGCCTTCACTGTCTGCACCAC -3^′^	
Integrin β3	F: 5^′^-TTCAATGCCACCTGCCTCAA-3^′^	98
	R: 5^′^-TTGGCCTCAATGCTGAAGCTC-3^′^	
Integrin β1	F: 5^′^-CAAGCAGGGCCAAATTGTGG-3^′^	185
	R: 5^′^-CCTTTGCTACGGTTGGTTACATT-3^′^	
ILK	F: 5^′^- CCAATGTCCTGGTCGCATGTA -3^′^	132
	R: 5^′^- CGTGTCACCAGTTCCCACAGA -3^′^	
FAK	F: 5^′^-CTGGCAGCATCTATCCAGGTCA-3^′^	144
	R: 5^′^-TTGGCAACACTTGCCCAATC-3^′^	
P53	F: 5^′^- AACGGTACTCCGCCACC-3^′^	94
	R: 5^′^- CGTGTCACCGTCGTGGA-3^′^	
P21	F: 5^′^- CACTCAGAGGAGGAAAATCCAGT −3^′^	90
	R: 5^′^- TTCTGACATGGCGCCTGCCT −3^′^	
Cyclin D1	F: 5^′^-CCGAGAAGCTGTGCATCTACAC-3^′^	94
	R: 5^′^-AGGTTCCACTTGAGCTTGTTCAC-3^′^	
PCNA	F: 5^′^- CTGTAGCGGCGTTGT -3^′^	133
	R: 5^′^- ACTTTCTCCTGGTTTGG -3^′^	
Bcl-2	F: 5^′^- TCAGGGACGGGGTGAACT -3^′^	143
	R: 5^′^- CAGGTGCCGGTTCAGGTACTC -3^′^	
Bax	F: 5^′^- CGCCGTGGACACAGACTC -3^′^	108
	R: 5^′^- GCAAAGTAGAAAAGGGCGACAAC -3^′^	
u-PA	F: 5^′^-TCTGCCTGCCCTCGATGTATAAC-3^′^	179
	R: 5^′^-GGTGGTGACTTCAGAGCCGTAGTAG-3^′^	
PAI-1	F: 5^′^-GGTCTCCAAACCAGACGGTGA-3^′^	188
	R: 5^′^-TGGCAATGTGACTGGAACAGAAATA-3^′^	
uPAR	F: 5^′^- ATCACCAGCCTTACCGAGGTTG -3^′^	87
	F: 5^′^- ACGGCTTCGGGAATAGGTGAC -3^′^	
MMP2	F: 5^′^-TGACATCAAGGGCATTCAGGAG-3^′^	134
	R: 5^′^-TCTGAGCGATGCCATCAAATACA-3^′^	
MMP9	F: 5^′^- CGCCCATTTCGACGATGAC -3^′^	80
	R: 5^′^- CGCCATCTGCGTTTCCAA -3^′^	
TIMP1	F: 5^′^- ACAGACGGCCTTCTGCAATTC-3^′^	166
	R: 5^′^- GGTGTAGACGAACCGGATGTCA -3^′^	
TIMP2	F: 5^′^- GTTCAAAGGGCCTGAGAAGGA -3^′^	166
	R: 5^′^- CCAGGGCACGATGAAGTCA-3^′^	
GAPDH	F: 5- GGGAAACTGTGGCGTGAT -3^′^	299
	R: 5^′^- GAGTGGGTGTCGCTGTTGA -3^′^	

### Western blot

Cells were lysed in RIPA buffer containing 1mM PMSF. Fifty microgram of protein per lane was resolved by SDS-PAGE, transferred to PVDF membrane and blocked with 5% BSA. The membranes were first incubated with primary antibody against SPARC (AF941, R&D Systems), PCNA (bs-0754R, BIOSS), cyclin D1 (bs-0623R, BIOSS), P53 (bs-0033R, BIOSS), P21 (bs-0741R, BIOSS), Bax (bs-0127R, BIOSS) and Bcl-2 (bs-0032R, BIOSS) at 1:1000 dilutions overnight, and then incubated with secondary antibody horseradish peroxidase-conjugated anti-goat or anti-rabbit IgG for 1 hour at room temperature. Blots were developed using ECL method and band intensity was analyzed using Gel-Pro Analyzer Software (Media Cybernetics, Inc., Bethesda, MD).

### Immunocytochemistry (ICC)

Logarithmic growth phase cells were seeded into 6-well plate covered with coverslips inside, after 24 h culture, coverslips were collected and subjected to Immunocytochemistry (ICC). ICC was performed according to streptavidin-biotin-peroxidase complex procedures. The coverslips were incubated with anti-human SPARC antibody (AF941, R&D Systems) with working dilution 15μg/ml at 4°C overnight. The secondary antibody was horseradish peroxidase conjugated anti-goat IgG. A negative control was obtained by replacing the primary antibody with normal rabbit immunoglobulin (IgG). Positive expression of SPARC was defined as the presence of brown granules in the cytoplasm. The intensity of staining and the distribution of positive cells were used to evaluate SPARC expression.

### Cell proliferation assays

Cell proliferation was determined by MTT assay and anchorage independent soft agar colony formation assay. For MTT assay, 20μl MTT (5 mg/ml in PBS) was added directly into each well of 96-well plate and incubated at 37°C for 4 h. Then media was removed and 200μl DMSO was added to dissolve formazan crystals. The optical density at 570 nm was read by a microplate reader (Molecular Devices, Sunnyvale, CA).

For soft agar colony formation assay, 500 μl 2×DMEM supplemented with 20% FBS was mixed with 500 μl 1.2% Sea Plague agar and solidified in each well of a 24-well plate to form base agar layer. For top agar layer, 25 μl cells (5×10^3^/ml) were mixed with 500 μl 2×DMEM and 500 μl 0.7% Sea Plague agar and added on top of base agar layer. After grown for 14 days, colony formation was monitored through a microscope. A cluster of ten cells or more was defined as a colony.

### Annexin V-PI assays for apoptosis

Cells were collected and washed twice with PBS, suspended in 200 μl binding buffer and 10 μl Annexin V-FITC for 20 minutes in the dark, and thereafter, 300 μl binding buffer and 5 μl propidium iodide (PI) were added to each sample. The apoptotic cells were determined by a flow cytometer (Becton Dickinson) with CellQuest (Becton Dickinson) software.

### Analysis of cell cycle distribution

Cells were harvested, washed with ice-cold PBS, and stained with 50 μg/ml PI and 250 μg/ml RNase for 30 minutes. The percentage of cells in each phase of the cell cycle was determined by a computer-programmed ModFit LT2.0 DNA assay (Becton Dickinson) using a flow cytometer.

### Cell invasion assay and migration assay

Invasion assay was performed as described previously [10]. Each Transwell (BD Biosciences, Bedford, MA) was coated with 50 μl 1:3 dilution of Matrigel. Cells (0.2 × 10^6^) were resuspended in 200 μl serum-free media and seeded into the upper chamber. Conditioned media of NIH3T3 cell culture was filtered and added to the lower chamber as a chemotactic factor. After 12 h, non-invading cells remaining on the upper surface were removed, and cells on the lower surface were fixed, stained with hematoxylin and eosin (H&E), and counted. Cell migration assay was also performed using Transwells without Matrigel coating. Each experiment was performed at least in triplicate. Invading cell numbers of three Boyden chambers were counted and the data were expressed as mean ± SE.

### Tumor xenografts in nude mice

BALB/C-nu/nu 5-week-old female nude mice were purchased from National Resource Center for Rodent Laboratory Animal of China. Four nude mice were injected subcutaneously with 5.0×10^6^ cells, and eight nude mice were injected through the tail veins with the same cells once a week for 3 consecutive weeks. The mice were maintained in a sterile animal facility and monitored for tumor growth. The volumes of tumors were monitored at the indicated times and calculated according to the formula: 0.5 × length ×width^2^. After 2 months, the mice injected subcutaneously were killed and the tumors were dissected and measured to calculate the volume. After 3 months, the nude mice injected through the tail veins were killed and the lungs were dissected. Paraffin sections of the lung tissues were made, stained with hematoxylin and eosin (H&E) and observed through a microscope. The average values were expressed as mean ± SE. This animal experiment was approved by the Institutional Animal Care and Use Committee of Shandong University and was in compliance with all regulatory guidelines.

### Flow cytometry analysis

Cells at log phase were collected and adjusted to a density of 1-5×10^6^/ml with PBS, and then stained with 20μl fluorescein isothiocyanate (FITC)-conjugated monoclonal antibodies for 30 min at 25°C. Finally, the stained cells were analyzed by a flow cytometer. Data analysis was carried out using the program WinMDI. The test was performed in quadruplicate.

### Zymography

The zymography assay was performed in 10% SDS-PAGE gel containing 0.1 mg/ml gelatin. 20μg protein sample was loaded into each lane of SDS-PAGE gel. After electrophoresis, the gel was washed twice with 2.5% Triton X-100 for 1h at room temperature to remove SDS and incubated at 37°C for overnight in the reaction buffer. After staining with Coomassie brilliant blue, MMPs expressions were identified as clear zones against the blue background.

### Statistical analysis

The results of cell and molecular biology data are expressed as the mean ± SE. For comparison of means between two groups, a two-tailed t-test was used, and for comparison of means among three groups, one-way ANOVA was used. Statistical analysis was performed using SPSS software version 13.0. P-value < 0.05 was considered statistically significant.

## Results

### Establishment of high invasive subclones HeLa-1 and SiHa-1 and low invasive subclones HeLa-25 and SiHa-23

**Figure 1:**
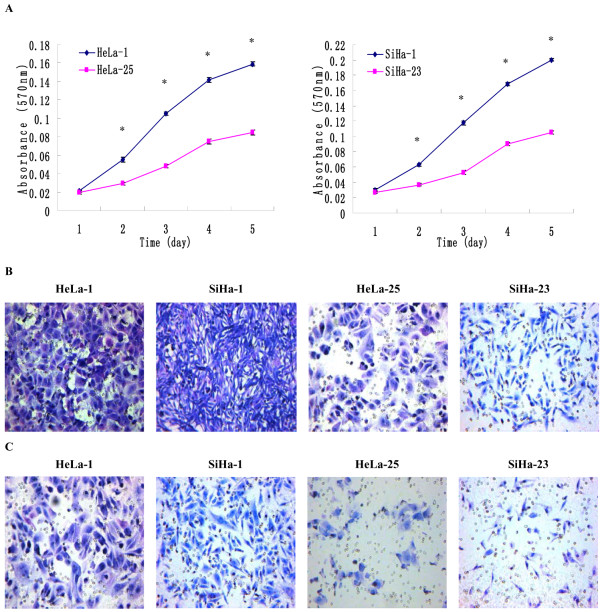
**Establishment of high invasive subclones and low invasive subclones.** (**A**) The proliferations of high invasive subclones HeLa-1 and SiHa-1 and low invasive subclones HeLa-25 and SiHa-23 as examined by MTT assay. (**B**) The images of cells crossing PVPF filters as examined by cell migration assay using Boyden chambers. (**C**) The images of cells invading Matrigel-coated membranes as examined by cell invasion assay using Boyden chambers. (Magnification ×200). *P<0.05 versus control.

**Figure 2:**
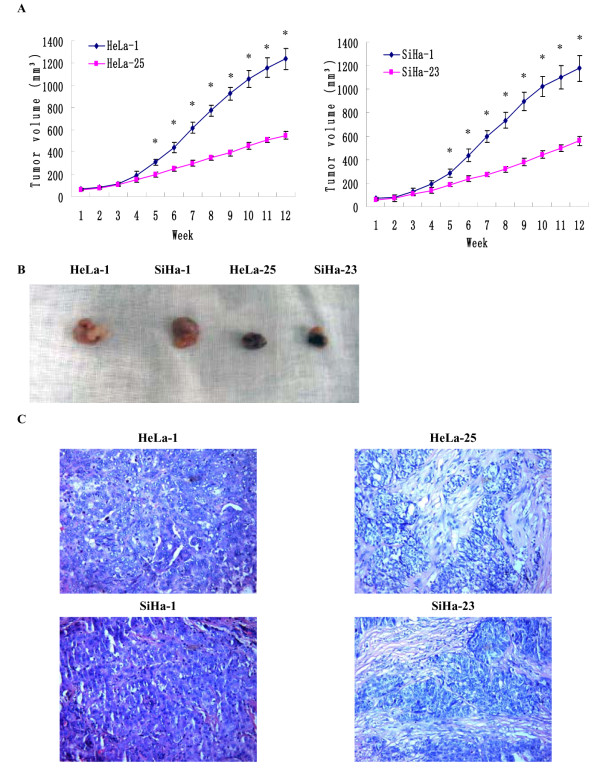
**Tumor xenografts in nude mice.** (**A**) Tumor growths of high invasive subclones HeLa-1 and SiHa-1 and low invasive subclones HeLa-25 and SiHa-23 observed continuously for 12 weeks. (**B**) Photograph of xenografts dissected from nude mice after subcutaneous inoculation. (**C**) H&E staining photos of xenografts dissected from nude mice after subcutaneous inoculation. (Magnification ×200). *P<0.05 versus control.

By the limited dilution method, a total of 25 subclones (HeLa-1-HeLa-25) and 23 subclones (SiHa-1-SiHa-23) were obtained from HeLa and SiHa cells. According to the electrophoretic migration rates, we selected HeLa-1 and SiHa-1 subclones that had the highest migration rates (18.23±0.31 μm/s and 19.54±0.38 μm/s), and HeLa-25 and SiHa-23 subclones that had the lowest migration rates (9.87±0.14 μm/s and 8.73±0.15 μm/s). HeLa-1 and SiHa-1 subclones showed higher proliferation and invasive activities than HeLa-25 and SiHa-23 subclones. The growth abilities in vitro of HeLa-1 and SiHa-1 subclones were higher than HeLa-25 and SiHa-23 subclones (Figure 1A). In the soft agar colony formation assay, the colony number formed by HeLa-1 and SiHa-1 subclones (45.73±1.14 and 48.57±1.25) was significantly higher than that formed by HeLa-25 and SiHa-23 subclones (5.32±0.28 and 6.73±0.35, P<0.01). In the cell migration assay (Figure 1B), the average migrating cell counts of HeLa-1 and SiHa-1 subclones were much higher than that of HeLa-25 and SiHa-23 subclones (218.48±4.75 and 265.24±5.36 vs. 76.64±3.41 and 85.91±3.87, P<0.01). In the Matrigel invasion assay (Figure 1C), the average invading cell counts of HeLa-1 and SiHa-1 subclones were much higher than that of HeLa-25 and SiHa-23 subclones (120.48±3.87 and 128.57±4.31 vs. 28.21±1.54 and 30.78±1.87, P < 0.01). In the transplantation assay (Figure 2AB), the tumor-forming rates of HeLa-1 and SiHa-1 subclones were 100% and that of HeLa-25 and SiHa-23 subclones were only about 50%. The volumes of tumors formed by HeLa-1 and SiHa-1 subclones were 14625.48±125.37 mm^3^ and 13167.25±128.68 mm^3^, much larger than that formed by HeLa-25 and SiHa-23 subclones (343.54±21.86 mm^3^ and 487.35±28.75 mm^3^, P<0.01). The results of H&E staining showed that the tumor xenografts formed by the low invasive subclones HeLa-25 and SiHa-23 consisted of more connective tissues and less tumor tissues, compared to that formed by the high invasive >subclones HeLa-1 and SiHa-1 (Figure 2C). HeLa-25 and SiHa-23 groups had no lung metastasis; however, the lung metastasis rates of HeLa-1 and SiHa-1 groups were about 70%. In conclusion, HeLa-1 and SiHa-1 subclones have the higher invasive potential in contrast with HeLa-25 and SiHa-23 subclones.

### The expression of SPARC in high invasive subclones HeLa-1 and SiHa-1 and low invasive subclones HeLa-25 and SiHa-23

**Figure 3:**
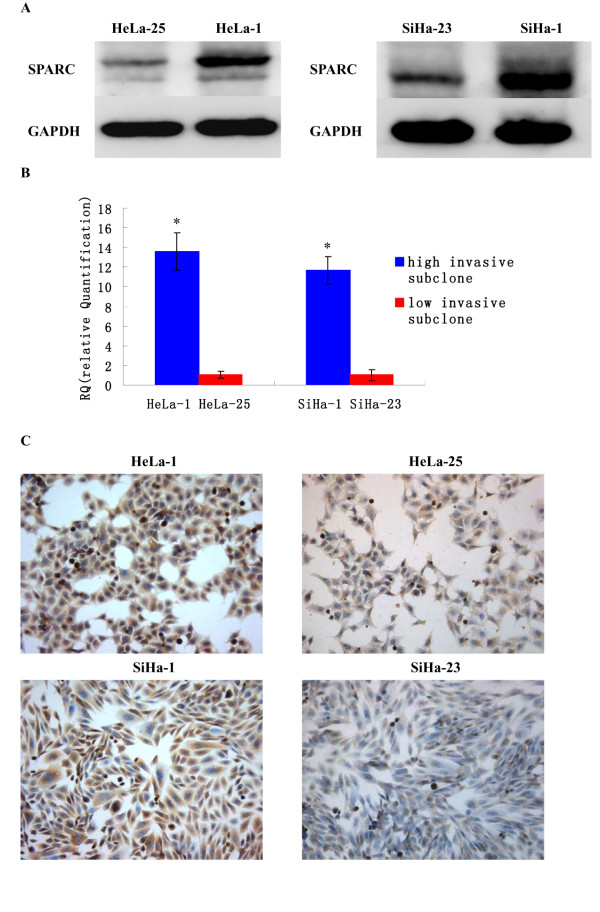
**Expression of SPARC in high invasive subclones and low invasive subclones.** (**A**) SPARC protein expressions of high invasive subclones HeLa-1 and SiHa-1 and low invasive subclones HeLa-25 and SiHa-23 as measured by Western blot. (**B**) SPARC mRNA expressions of high invasive subclones HeLa-1 and SiHa-1 and low invasive subclones HeLa-25 and SiHa-23 as measured by q-RT-PCR. (**C**) SPARC protein expressions of high invasive subclones HeLa-1 and SiHa-1 and low invasive subclones HeLa-25 and SiHa-23 as measured by ICC staining (Magnification ×200). *P<0.05 versus control.

We know that SPARC is associated with the progressions of many human tumors. However, till now few researches have been done about the functions of SPARC in human cervical cancer cell growth and metastasis. So we detect the different expressions of SPARC between high invasive subclones HeLa-1 and SiHa-1 and low invasive subclones HeLa-25 and SiHa-23. The results of Western Blot (Figure 3A) showed that the average band intensities of SPARC normalized to GAPDH in HeLa-1 and SiHa-1 groups were 0.97±0.15 and 1.38±0.14, much higher than that in HeLa-25 and SiHa-23 groups (0.16±0.04 and 0.35±0.07, P<0.01). Real-time RT-PCR and ICC (Figure 3BC) also revealed that the expressions of SPARC were much higher in high invasive subclones HeLa-1 and SiHa-1 than that in low invasive subclones HeLa-25 and SiHa-23.

### Knockdown of SPARC expression by lentivirus-mediated RNA interference

**Figure 4:**
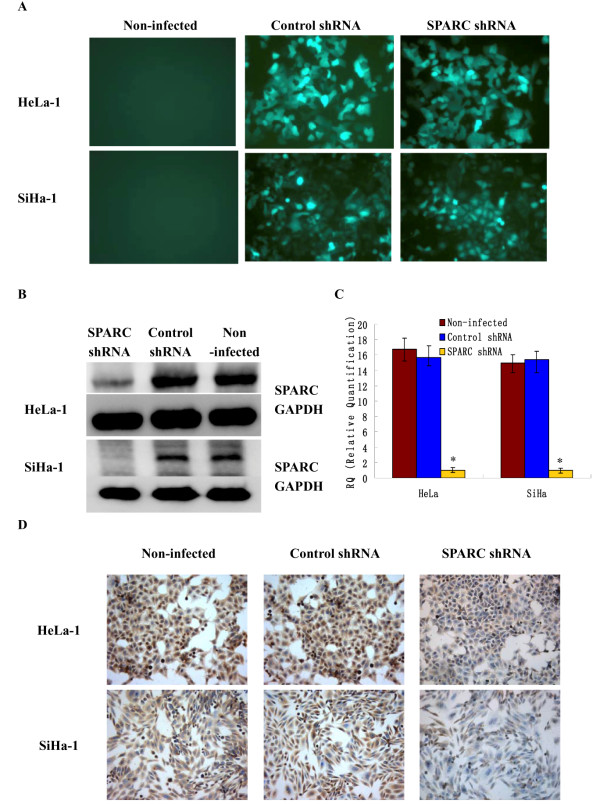
**Verification of knockdown of SPARC expression by lentivirus-mediated RNA interference.** (**A**) GFP expression images showed shRNA delivery efficiency. (Magnification × 200). (**B**) SPARC protein expressions of SPARC shRNA infected cells, control shRNA infected cells and non-infected cells as measured by Western blot. (**C**) SPARC mRNA expressions of SPARC shRNA infected cells, control shRNA infected cells and non-infected cells as measured by q-RT-PCR. (**D**) SPARC protein expressions of SPARC shRNA infected cells, control shRNA infected cells and non-infected cells as measured by ICC staining (Magnification ×200). *P<0.05 versus control.

To investigate the role of SPARC in cervical cancer, we constructed lentivirus vector with SPARC shRNA and infected high invasive subclones HeLa-1 and SiHa-1. After viral infection, more than 80% cells were GFP-positive, indicating a high efficiency of shRNA delivery (Figure 4A). The results of Western Blot (Figure 4B) showed that the average band intensities of SPARC normalized to GAPDH in SPARC shRNA infected group, control shRNA infected group and non-infected group were separately 0.14±0.05, 0.90±0.17 and 0.92±0.13 for HeLa-1 subclone, and 0.07±0.02, 0.76±0.09 and 0.80±0.07 for SiHa-1 subclone. As shown in Figure 4C-D, Real-time RT-PCR and ICC also confirmed the down-regulation of SPARC by its shRNA. There was no significant difference between control shRNA infected cells and non-infected cells. SPARC expressions were decreased in high invasive subclones HeLa-1 and SiHa-1 at both mRNA and protein levels after RNA interference.

### Knockdown of SPARC expression suppressed cervical cancer cell proliferation

**Figure 5:**
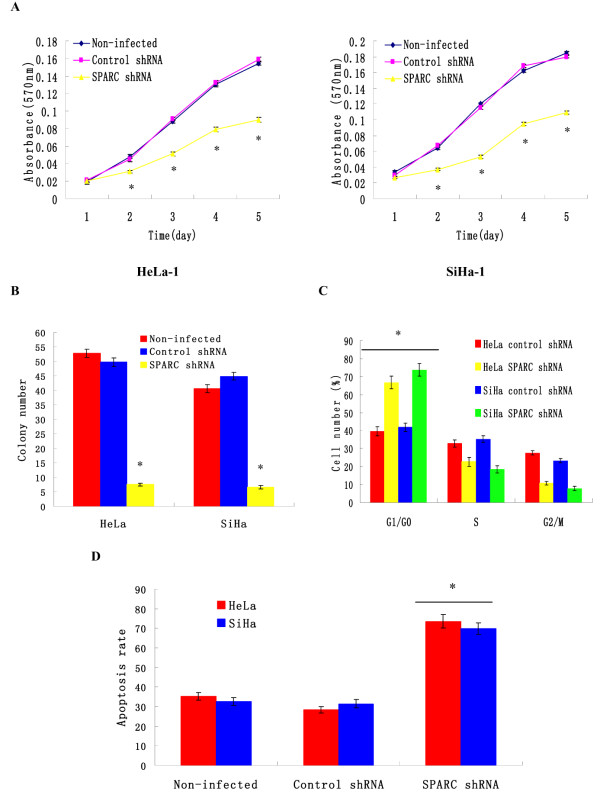
**Effects of SPARC knockdown on cell growth, colony formation and apoptosis.** (**A**) Cell proliferations of SPARC shRNA infected cells, control shRNA infected cells and non-infected cells as examined by MTT assay. (**B**) Anchorage independent growth of SPARC shRNA infected cells, control shRNA infected cells and non-infected cells as measured by soft agar colony formation assay. (**C**) Cell-cycle distributions of SPARC shRNA infected cells and control shRNA infected cells as measured by flow cytometry. (**D**) Cell apoptosis of SPARC shRNA infected cells, control shRNA infected cells and non-infected cells as measured by Annexin V-PI assays. *P<0.05 versus control.

We investigated the effects of SPARC knockdown on the proliferations of both high invasive subclones HeLa-1 and SiHa-1. MTT results showed that SPARC knockdown significantly reduced cell proliferations of HeLa-1 and SiHa-1 (Figure 5A). In the soft agar colony formation assay, HeLa-1 and SiHa-1 cells infected with SPARC shRNA showed significant reduction in the colony formation (Figure 5B). No significant difference was found between control shRNA infected cells and non-infected cells.

### Knockdown of SPARC expression induced cell cycle arrest at the G1/G0 phase

To understand the mechanism of SPARC in cervical cancer cell proliferation, we used flow cytometry to identify the specific phases of the cell cycle. As shown in Figure 5C, HeLa-1 and SiHa-1 cells infected with SPARC shRNA contained 20-30% more cells at the G1 or G0 (G1/G0) phase (*P* < 0.01), compared with the control shRNA infected cells. These data indicated that knockdown of SPARC expression inhibited the proliferation of cervical cancer cells by blocking their progression from the G1/G0 phase to the S phase during the cell cycle.

### Knockdown of SPARC expression induced cervical cancer cell apoptosis

As shown in Figure 5D, the percentage of apoptotic cells infected with SPARC shRNA was much higher than that in control shRNA group (*P* < 0.01). No significant difference was found between control shRNA infected cells and non-infected cells. These data indicated that knockdown of SPARC expression induced cervical cancer cell apoptosis.

### Knockdown of SPARC expression inhibited cervical cancer cell migration and invasion

**Figure 6:**
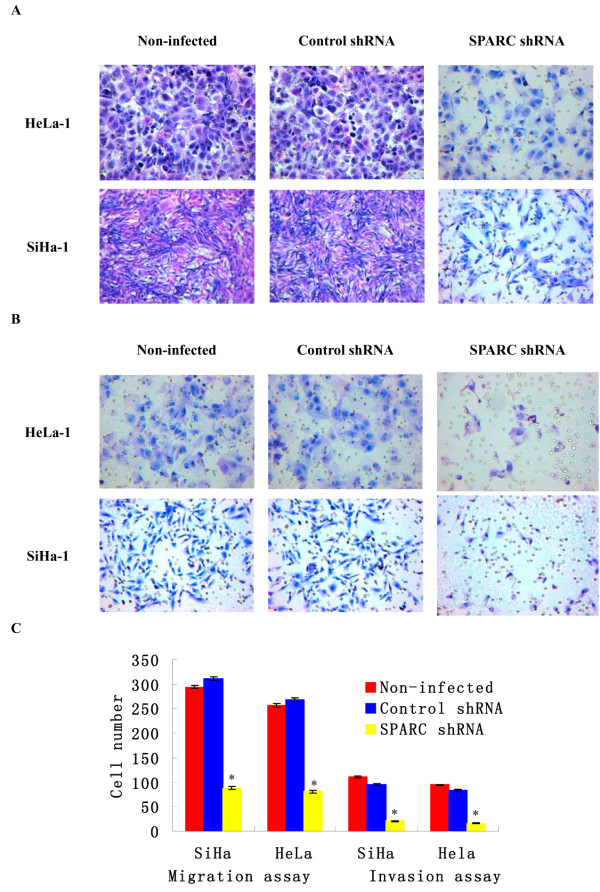
**Effects of SPARC knockdown on cell migration and invasion.** (**A**) The images of cells migrating PVPF filters as examined by cell migration assay using Boyden chambers. (**B**) The images of cells invading Matrigel-coated membranes as examined by cell invasion assay using Boyden chambers. (Magnification ×200). (**C**) The average invading or migrating cell counts of SPARC shRNA infected cells, control shRNA infected cells and non-infected cells. *P<0.05 versus control.

We further examined the effects of SPARC knockdown on the migration and invasion abilities of HeLa-1 and SiHa-1 cells. As shown in Figure 6, knockdown of SPARC inhibited cervical cancer cells invasion and migration. The similar data were achieved in HeLa-1 and SiHa-1 cells after SPARC shRNA infections. The average invading or migrating cell count of SPARC shRNA infected cells was much less than that of control shRNA infected cells. No significant difference was found between control shRNA infected cells and non-infected cells.

### Knockdown of SPARC expression inhibited tumor growth and lung metastasis in nude mice

**Figure 7:**
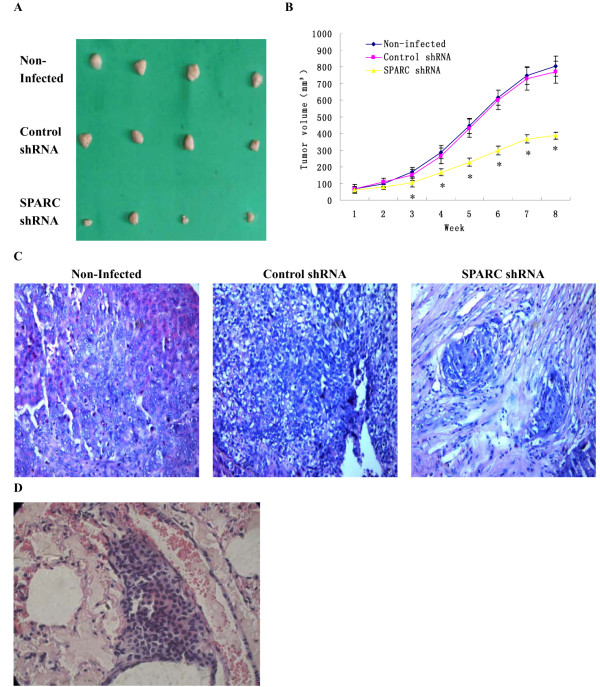
**Effects of SPARC knockdown on tumor growth and lung metastasis in nude mice.** (**A**) Photograph of xenografts dissected from nude mice after subcutaneous inoculation (n=4). (**B**) Tumor growths of SPARC shRNA infected cells, control shRNA infected cells and non-infected cells observed continuously for 8 weeks. (**C**) H&E staining photos of xenografts dissected from nude mice after subcutaneous inoculation. (**D**) Photograph of lung metastasis through a microscope after inoculation through tail vein. (n=8) (Magnification ×200). *P<0.05 versus control.

Tumor formation was performed in high invasive subclone HeLa-1 cells infected by SPARC shRNA. Cells were inoculated subcutaneously in nude mice and tumor growth was measured after 2 months. As shown in Figure 7AB, knockdown of SPARC showed a decrease in the size of tumors, compared with its control counterpart. The results of H&E staining showed that the tumor xenografts formed by SPARC shRNA infected cells consisted of more connective tissues and less tumor tissues (Figure 7C). There was no significant difference between control shRNA infected cells and non-infected cells in the tumor formation of nude mice. Moreover, Figure 7D shows the lung metastasis through a microscope after tail vein injection into nude mice. About 50% lung metastases were found after 3 months in the nude mice injected with control shRNA infected cells, and the average lung colony size was 267.84±12.68 mm^3^, while no lung metastasis was found after injection with SPARC shRNA infected cells into nude mice.

### Downstream molecules of SPARC in cervical cancer cell

**Figure 8:**
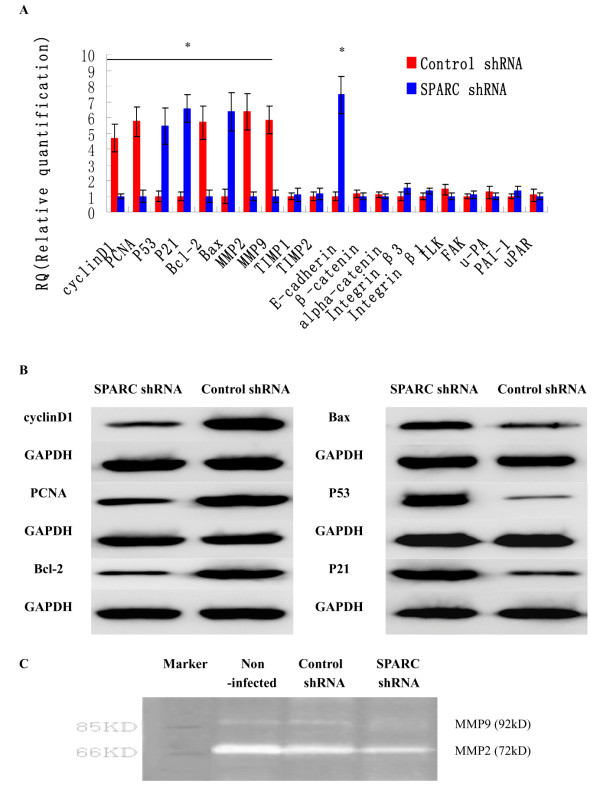
**Downstream target genes of SPARC in cervical cancer cells.** (**A**) E-cadherin, β-catenin, alpha-catenin, Integrin β3, Integrin β1, ILK, FAK, P53, P21, Cyclin D1, PCNA, Bcl-2, Bax, u-PA, uPAR, PAI-1, MMP2, MMP9, TIMP1 and TIMP2 mRNA expressions as measured by q-RT-PCR. (**B**) P53, P21, Cyclin D1, PCNA, Bcl-2 and Bax protein expressions in SPARC shRNA infected cells and control shRNA infected cells as measured by Western blot. (**C**) MMPs expressions of SPARC shRNA infected cells, control shRNA infected cells and non-infected cells as measured by zymography. *P<0.05 versus control.

To understand the mechanism of SPARC in cervical cancer cell proliferation, apoptosis and invasion, the downstream molecules of SPARC were detected by real-time quantitative RT-PCR in high invasive subclone HeLa-1 cells. As shown in Figure 8AB and Table 2, Cyclin D1, PCNA, Bcl-2, MMP2 and MMP9 were significantly down-regulated in SPARC shRNA infected cells, the average band intensities of Cyclin D1, PCNA, and Bcl-2 normalized to GAPDH in SPARC shRNA infected group were separately 0.26±0.04, 0.36±0.03 and 0.21±0.04, much lower than that in control shRNA infected group (1.14±0.13, 1.26±0.15 and 0.96±0.10). Besides, higher expression levels of E-cadherin, P53, P21 and Bax were found in SPARC shRNA infected cells, the average band intensities of P53, P21 and Bax normalized to GAPDH in SPARC shRNA infected group were separately 0.89±0.11, 0.93±0.12 and 1.24±0.16, much higher than that in control shRNA infected group (0.24±0.03, 0.16±0.02, and 0.38±0.08). There were no different expressions of β-catenin, α-catenin, Integrin β3, Integrin β1, ILK, FAK, u-PA, PAI-1, uPAR, TIMP1 and TIMP2 between SPARC shRNA infected cells and control shRNA infected cells. Zymography results showed that the MMPs expressions were significantly reduced in SPARC shRNA infected cells (Figure 8C).

**Table 2:** Flow cytometry analysis about E-cadherin, integrin β1 and integrin β3

	**Percent positive cells**		***P***
	**SPARC shRNA**	**Control shRNA**	
E-cadherin	80.38±5.73	24.85±2.64	<0.01
Integrin β1	95.35±6.67	97.43±8.12	>0.05
Integrin β3	53.78±3.37	55.67±3.62	>0.05

## Discussion and conclusions

SPARC is a highly conserved, multifunctional matricellular protein that regulates matrix remodeling, cell adhesion and migration, cell cycle regulation and angiogenesis. Although there is growing evidence for an important role for SPARC in a variety of cancers, there is no unifying model, which explains all aspects of its function [9,11]. For example, higher levels of SPARC expression have been reported in breast cancer [12,13], melanoma [14,15], hepatocellular carcinoma [16,17], prostate cancer [18] and colorectal cancer [19,20]. However, the opposite effect has also been demonstrated, suggesting that SPARC may be able to inhibit tumorigenesis or tumor progression in breast cancer [21,22], ovarian carcinoma [23,24], hepatocellular carcinoma [25], prostate cancer [26], and colorectal cancer [27,28]. In cervical cancer, recent researches reveal that increased expression of SPARC is related to the progression of cervical cancer and often accompanied with aberrant methylation [29,30]. In our study, we found that SPARC was highly expressed in the high invasive subclones HeLa-1 and SiHa-1, compared to the low invasive subclones HeLa-25 and SiHa-23. These results revealed that SPARC was related to the invasive phenotype of cervical cancer cells. Further researches showed that lentivirus-mediated knockdown of SPARC expression significantly suppressed cervical cancer cell proliferation, induced cell apoptosis, and inhibited cell invasion and metastasis.

In this study, using MTT assay, soft agar colony formation assay and flow cytometer, we concluded that knockdown of SPARC suppressed cervical cancer cell proliferation. Flow cytometer showed that knockdown of SPARC reduced the number of cells in S-phase, while increased the number of cells in G1/G0-phase, indicating G1/G0 arrest. To understand the mechanism of SPARC in cervical cancer cell proliferation, we detected the different expressions of Cyclin D1, PCNA, P53 and P21 between SPARC shRNA infected cells and control shRNA infected cells. Cyclin D1, a member of G1 cyclins, and PCNA (proliferating cell nuclear antigen) are proliferation biomarkers [31,32]. P53, a tumor suppressor gene, and P21 (cyclin-dependent kinase inhibitor), a key mediator of p53, can induce cell-cycle arrest in the G1-S checkpoint [33,34]. In this study, we found that knockdown of SPARC expression could decrease the expressions of Cyclin D1 and PCNA and increase the expressions of P53 and P21, which suggested that depletion of SPARC could inhibit cervical cancer cell proliferation through activation of a p53⁄p21^Cip1⁄Waf1^ pathway dependent on G1-S checkpoint. In melanoma, SPARC also could regulate cell cycle progression and proliferation through the p53/p21 (Cip1/Waf1) pathway [35]. In brief, SPARC could play an important role in cervical cancer cell proliferation by controlling cell cycle progression, but how depletion of SPARC can lead to activation of the p53/p21 (Cip1/Waf1) signaling pathway needs further study.

Further researches showed that knockdown of SPARC induced cervical cancer cell apoptosis. Apoptosis is modulated partially by Bcl-2 family including apoptosis-inhibiting genes (Bcl-2, Bcl-xL, Mcl-1, A1 and Bcl-w) and apoptosis-accelerating genes (Bax, Bak, Bcl-xS and Bim) [36]. In this study, we found that knockdown of SPARC could decrease the expression of apoptosis-inhibiting gene Bcl-2 and increase the expression of apoptosis-accelerating gene Bax, indicating the down-regulated ratio of Bcl-2/Bax. Our result revealed that SPARC could play an effect on apoptosis by changing the ratio of Bcl-2/Bax. Similar results were found in human melanoma; suppression of SPARC in several human melanoma cells triggered apoptotic cell death dependent on p53 and induction of Bax [37]. Apoptosis, programmed cell death, is vital for normal development and tissue homeostasis. Our data suggested that SPARC as an antistress factor could promote cervical cancer cell survival through suppression of apoptotic pathways.

Using Boyden chambers and xenografts in nude mice, we concluded that knockdown of SPARC expression inhibited cervical cancer cell invasion and metastasis. To clarify the mechanism of SPARC in cervical cancer cell invasion and metastasis, after viral infection, we detected the expression of the cellular adhesion molecule E-cadherin and integrins, which mediated cell-cell adhesion and cell-extracellular matrix adhesion, and the expression of proteolytic enzymes such as plasminogen activator/plasmin system (uPA-uPAR) and matrix metalloproteinases (MMPs), which degraded the extracellular matrix (ECM). These results revealed that knockdown of SPARC up-regulated the expression of E-cadherin and had no effect on the expressions of integrin β1 and integrin β3. SPARC can break down cell-cell connections to improve tumor invasion by changing E-cadherin expression. Similar results were also found in melanoma [38,39]; SPARC can down-regulate E-cadherin and stimulate an invasive melanoma phenotype. Next, knockdown of SPARC decreased the expressions of MMP2 and MMP9, but no significant differences were found in the expressions of u-PA, uPAR, PAI-1, TIMP1 and TIMP2 between SPARC shRNA infected cells and control shRNA infected cells. SPARC can make the extracellular matrix degradation by MMP2 and MMP9. Similar results were also found in glioma [40,41]. Our data suggested that depletion of SPARC could promote the homophilic cell-cell adhesion by up-regulating E-cadherin and restrained extracellular matrix degradation by down-regulating MMPs expressions to inhibit cervical cancer cell invasion and metastasis.

In conclusion, SPARC is associated with cervical cancer cell growth and metastasis. Knockdown of SPARC expression significantly suppresses cervical cancer cell proliferation, induces cell apoptosis and inhibits cell invasion and metastasis. All of these informations contribute to a better understanding that SPARC, as a promoter, improves cervical cancer cell proliferation, invasion and metastasis.

## Competing interests

The authors declare that they have no competing interests.

## Authors’ contributions

XL performed the limited dilution method. JC performed the siRNA transfections. SF and JC performed ICC, real time RT-PCR and western blot experiments. JZ and JC performed the function assays and conceived the study and drafted the manuscript. DS and YZ participated in the design and coordination of the study. All authors have read and approved the final manuscript.

## Pre-publication history

The pre-publication history for this paper can be accessed here:

http://www.biomedcentral.com/1471-2407/12/464/prepub
